# Screening and Validation of Leaf Width-Related Genes in Inbred Maize Lines

**DOI:** 10.3390/life14091057

**Published:** 2024-08-23

**Authors:** Shi Lu, Qi Wang, Junqi Yin, Shubo Zheng, Tingting Gao, Xudong Zhou, Jianxin Zhang, Yuexian Xing, Yingjie Ma, Min Wang, Delong Zhou, Ming Lu, Wenguo Liu, Piwu Wang, Zhijun Zhang

**Affiliations:** 1 Jilin Academy of Agricultural Sciences (Northeast Innovation Center of Agricultural Science and Technology in China), Shengtai Street, No.1363, Changchun 130033, China; 2 Jilin Jinong Hi-tech Inc., Ltd., Kemao Street, No.303, Gongzhuling 136100, China; 3 College of Agronomy, Jilin Agricultural University, Xincheng Street, No.1288, Changchun 130118, China

**Keywords:** maize (*Zea mays* L.), leaf width, GWAS, *Zm00001d044327*, qPCR analysis, EMS mutants

## Abstract

Leaf width is a key determinant of planting density and photosynthetic efficiency. In an effort to determine which genes regulate maize plant leaf width, we performed a genome-wide association study (GWAS) of 1.49 × 106 single nucleotide polymorphisms (SNPs) in 80 sequenced backbone inbred maize lines in Jilin Province, China, based upon phenotypic leaf width data from two years. In total, 14 SNPs were identified as being significantly related to leaf width (*p* < 0.000001), with these SNPs being located on chromosomes 1, 2, 3, 5, 6, 7, 8, and 9. A total of five candidate genes were identified within a mean linkage disequilibrium (LD) distance of 9.7 kb, with a significant SNP being identified within the *Zm00001d044327* candidate gene. RNA was then isolated from 12 different inbred maize lines from this GWAS study cohort and was used to conduct qPCR analyses which revealed significant differences in *Zm00001d044327* expression among strains exhibiting significant differences in leaf width. Based on an assessment of EMS mutant lines harboring a conserved amino acid stop mutation and two non-synonymous mutations in *Zm00001d044327* that exhibited a narrow leaf width, these data suggested that *Zm00001d044327* is a key regulator of maize leaf width.

## 1. Introduction

Maize (Zea mays) is one of the most economically important crops globally, serving as a staple food, a feed for livestock, and a raw material in various industrial applications. As a cornerstone of agricultural economies, maize production significantly influences food security and economic stability in many countries. In commercial maize cultivation, a shorter growth period is preferred to reduce ear water content at harvest, which is critical for optimizing both grain quality and economic yield [1,2]. However, this can also diminish photosynthetic activity, adversely affecting plant health [3,4]. Consequently, a higher planting density is often employed to compensate and maintain high overall yields [5,6]. Leaf width is a primary characteristic of maize plants and plays a crucial role in these dynamics, serving as a central facet of density tolerance breeding strategies [7]. Quantitative trait loci (QTLs) associated with leaf width have been identified by various research groups using parental mapping strategies. For instance, Pelleschi et al. [8] identified three QTLs on chromosomes 1, 5, and 7 through restriction fragment length polymorphism (RFLP) analysis in an F_2_ × MBS847 population. Similarly, Ku et al. [9] reported five QTLs associated with leaf width on chromosomes 1, 2, 7, and 8, collectively explaining 34.13% of the phenotypic variance observed in this trait. These findings provide a foundational understanding of the genetic determinants of leaf width. 

Against this backdrop, our research team previously published a study in PLoS ONE [10], utilizing 1.49 million single nucleotide polymorphisms (SNPs) to sequence 80 maize inbred lines from Jilin Province. Based on phenotype data collected over two years, we conducted a genome-wide analysis of leaf angle and leaf orientation value. This study identified SNPs significantly associated with these critical traits, revealing that the regulatory genes for these traits hold potential importance in enhancing planting density and photosynthetic efficiency. In this study, we have further analyzed the genome-wide association study (GWAS) loci for leaf width, with a particular focus on genetic regions not covered in the PLOS One study, as well as new genes related to leaf width identified in this research. Our aim is to elucidate the genetic architecture of maize leaf width further, thereby providing valuable insights for the improvement of this economically significant crop species. 

Genome-wide association study (GWAS) strategies enable researchers to survey the genome in order to detect particular SNPs linked to traits of interest [11,12], permitting the high-resolution and high-throughput profiling of germplasm resources to select for key trait-associated alleles [13,14]. GWAS-based strategies have previously been employed to assess maize leaf architecture, with Tian et al. [15] and Buckler et al. [16] having utilized a nested association mapping (NAM) population in a joint linkage mapping approach that ultimately identified 34 leaf width-related QTLs. In addition, Wang et al. [17] detected the qLW4 region in the Huang C × X178 (H/X) population and found this region to be associated with leaf width. However, these prior studies did not perform any in-depth assessments of the candidate genes within their loci of interest. As such, we herein screened for leaf width-associated SNPs using 80 backbone inbred maize lines collected in Jilin Province, and we sought to assess the functional importance of detected genes in order to better understand maize genetics.

## 2. Materials and Methods

### 2.1. Study Population

A total of 80 parental inbred maize lines collected over a 5-year period from a >80% maize hybrid total planting area in Jilin Province were collected and utilized for the present GWAS analyses. All plants were planted in triplicate in a random block design in an experimental field in Changchun from 2015–2016. Leaf width measurements were conducted using a standard ruler.

### 2.2. Leaf Width Phenotypic Data Analysis

Leaf width was measured at the widest portion of the leaf above the uppermost ear in five plants during the R1 developmental stage, with the mean value being calculated. All statistical analyses of phenotypic data were conducted with SPSS v19.0 [18]. Statistical distinctions between samples were assessed using Student’s *t*-test (*p* ≤ 0.05).

### 2.3. Whole-Genome Sequencing

Whole-genome sequencing was conducted as described previously by Lu et al. [19]. Briefly, a modified CTAB approach was utilized to extract gDNA from all plants in the GWAS population [20]. Novogene Biological Company (Beijing, China) then conducted next-generation sequencing. For these sequencing analyses, samples were fragmented to yield 350 bp fragments via sonication, followed by A-tailing, end repair, and ligation to Illumina sequencing adapters. Following sequencing, adapters and ambiguous bases were removed from raw reads, followed by clean read alignment to the B73 maize genome (ZmB73_RefGen 4) with the BWA software (alignment via Burrows-Wheeler transformation) Version: 0. 5. 9-r16 [21,22]. SAMtools (v1.3) was utilized to remove duplicate reads with the rmdup parameters [23]. A miss rate of <10% and a minimum allele frequency (MAF) ≧ 0.05 were used to screen for high-quality SNPs associated with the 80 maize lines used in the present study.

### 2.4. GWAS

The present GWAS study was conducted via a fixed and random model Circulating Probability Unification [24] (FarmCPU) approach, with leaf width-associated SNPs being identified based upon a *p* < 0.000001 threshold. PLINK was used to calculate average LD decay distance values [24], with the B73 reference genome being used to identify candidate genes of interest within leaf width-related regions. Annotation and functional predictive analyses of known candidate genes were conducted with MaizeGDB and NCBI, while functional predictions for unknown genes were made based upon the Arabidopsis database TAIRP43 [25].

### 2.5. Candidate Gene Validation

After identifying *Zm00001d044327* as a candidate gene of interest harboring the significant sLW225305520 SNP, we employed a quantitative real-time PCR (qPCR) approach to assess the expression of this gene in 12 different inbred maize lines selected based upon their differences in leaf width phenotypes. These lines included those with broad leaves (W12, W62, W44, W38, W65, and W42) and those with narrow leaves (W56, W58, W03, W43, W48, and W36). When plants were in the silking stage (R1) of growth, total RNA samples were extracted and qPCR was employed to assess *Zm00001d044327* gene expression [26], with EF-1α as a normalization control. Primers used in the present analysis are shown in Table 1. We additionally obtained a mutant with one conserved amino acid termination mutation and two non-synonymous mutations in the LOG domain of *Zm00001d044327* in an EMS mutant library (http://www.elabcaas.cn/memd/index.php, accessed on 25 December 2023) as a means of verifying the functional relevance of this candidate gene.

## 3. Results

### 3.1. Study Population Phenotypic Characteristics 

**Figure 1:**
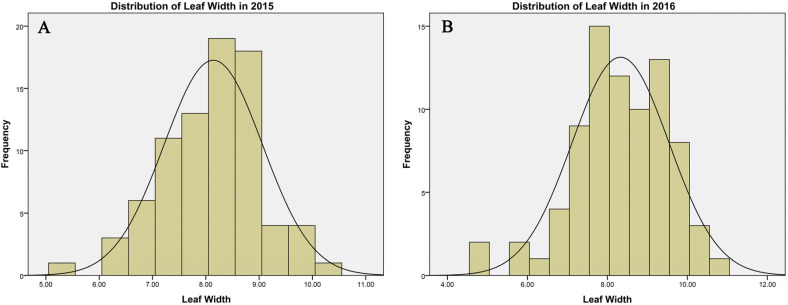
Distribution of leaf width in 2015 and 2016. (**A**) Phenotypic distribution of leaf width in 2015; (**B**) phenotypic distribution of leaf width in 2016.

The 80 inbred maize lines used for this GWAS analysis exhibited leaf width phenotypes conforming to a normal distribution over the course of this study (2015–2016) (Figure 1), with significant differences in width values among members of the study population (*p* < 0.01). Mean leaf width values in 2015 and 2016 were 8.1 and 8.3 cm, respectively (range: 4.8–10.8 cm).

### 3.2. NGS Data Quality and LD Analysis 

**Figure 2:**
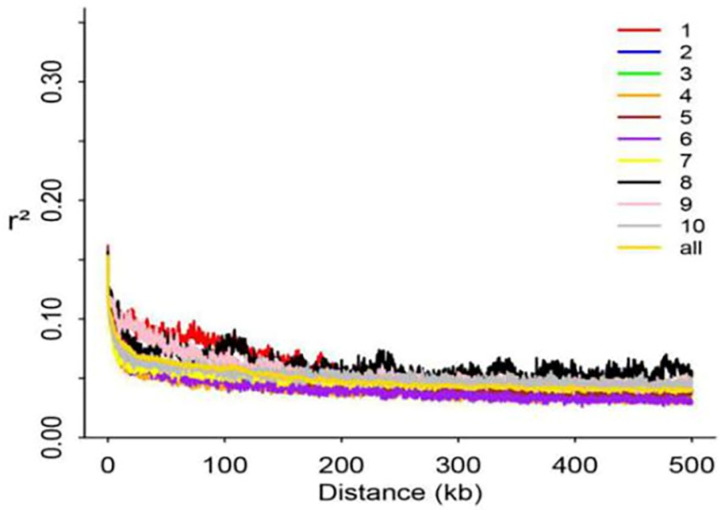
GWAS population LD decay map.

Next-generation sequencing of the study population generated 3230.75 GB of high-quality genomic data through approaches that have been detailed previously [18,19]. All sequence data from this study were uploaded to the Sequence Read Archive (https://www.ncbi.nlm.nih.gov/sra) under the accession number PRJNA495031. A total of 34,872,961 SNPs were identified in this analysis and filtered according to the criteria: MAF > 0.05, miss rate < 10%. This filtering and screening effort yielded 1,490,007 SNPs for subsequent analysis, with a mean population decay distance of 9.7 kb that was measured at an LD decay parameter (R2) of 0.1 (Figure 2).

### 3.3. Leaf Width-Related SNP Identification

**Figure 3:**
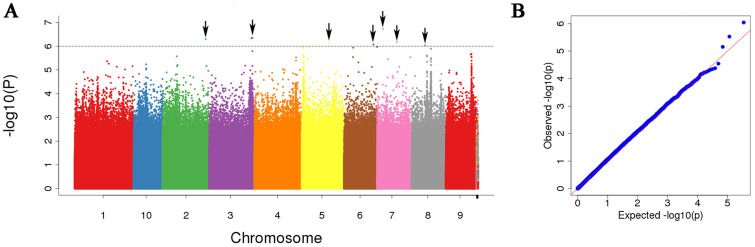
Manhattan plot and QQ plot of 2015 leaf width. (**A**) GWAS Manhattan plot of 2015 leaf width; (**B**) GWAS quantile–quantile (QQ) plot of 2015 leaf width.

**Figure 4:**
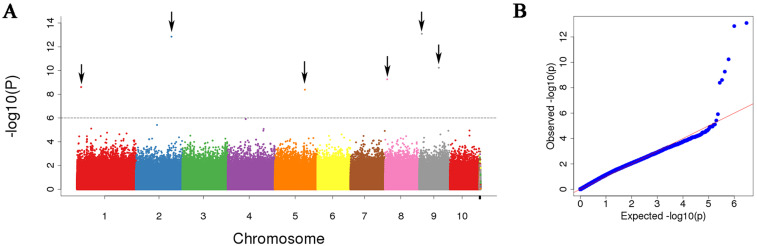
Manhattan plot and QQ plot of 2016 leaf width. (**A**) GWAS Manhattan plot of 2016 leaf width; (**B**) GWAS quantile–quantile (QQ) plot of 2016 leaf width.

In total, we identified 14 leaf width-related SNPs at a significance threshold of *p* < 0.000001 in the present GWAS analysis (Table 2), and we found these SNPs to be distributed on chromosomes 1, 2, 3, 5, 6, 7, 8, and 9 (Figure 3 and Figure 4). Eight of these SNPs, accounting for 0.4–13.2% of overall phenotypic variability, were detected in samples collected in 2015, whereas the other six SNPs, which accounted for 3.1–14.2% of overall phenotypic variability, were detected in samples collected in 2016. We localized the sLW225305520 marker to be within an exonic region of the *Zm00001d044327* candidate gene. 

### 3.4. Identification and Functional Annotation of Leaf Width-Related Candidate Genes

Scanning within the LD decay distance enabled us to identify five putative leaf width-associated candidate genes based upon the 14 SNPs identified in the above GWAS analysis. Of these genes, *Zm00001d044327*, which encodes a monophosphate phosphoribohydrolase, was found to harbor a significant SNP (sLW-225305520). Additionally, the 2310 bp *Zm00001d020287*, which encodes the hypothetical ZEAMMB73 protein, was 2528 bp away from a significant SNP (sLW-104304481; phenotype contribution rate = 7.1%). The 2539 bp *Zm00001d020285* candidate gene was predicted to code for the chloroplastic homolog 7 and was 3128 bp from sLW-104304481, while the 1739 bp *Zm00001d007267* candidate gene was predicted to encode a chlorophyll a-b binding protein and was 9706 bp from a significant SNP (sLW-226131901; phenotype contribution rate = 6.2%). 

### 3.5. Functional Confirmation of SNP-Associated Gene Variants

Given the presence of a significant SNP within the *Zm00001d044327* candidate gene, we next conducted a qPCR analysis to confirm the association between this SNP and differences in maize plant leaf width through comparative analyses. We additionally obtained a mutant with a conserved amino acid termination mutation and non-synonymous mutations in the LOG domain of *Zm00001d044327* to better validate the functional role of this gene.

### 3.6. qPCR Analysis of the Zm00001d044327 Candidate Gene

To better understand the relationship between *Zm00001d044327* and leaf width, we next chose six inbred maize lines with an average leaf width of <7 cm, including W56 (mean leaf width = 6.2 cm), W58 (mean leaf width = 6.3 cm), W3 (mean leaf width = 6.6 cm), W43 (mean leaf width = 6.8 cm), W48 (mean leaf width = 7 cm), and W36 (mean leaf width = 7 cm). We additionally selected six inbred lines with a mean leaf width > 9.3 cm, including W42 (mean leaf width = 9.3 cm), W65 (mean leaf width = 9.4 cm), W38 (mean leaf width = 9.4 cm), W44 (mean leaf width = 9.4 cm), W62 (mean leaf width = 9.5 cm), and W12 (mean leaf width = 9.7 cm). These 12 plant lines were then used in qPCR analyses to assess *Zm00001d044327* expression.

**Figure 5:**
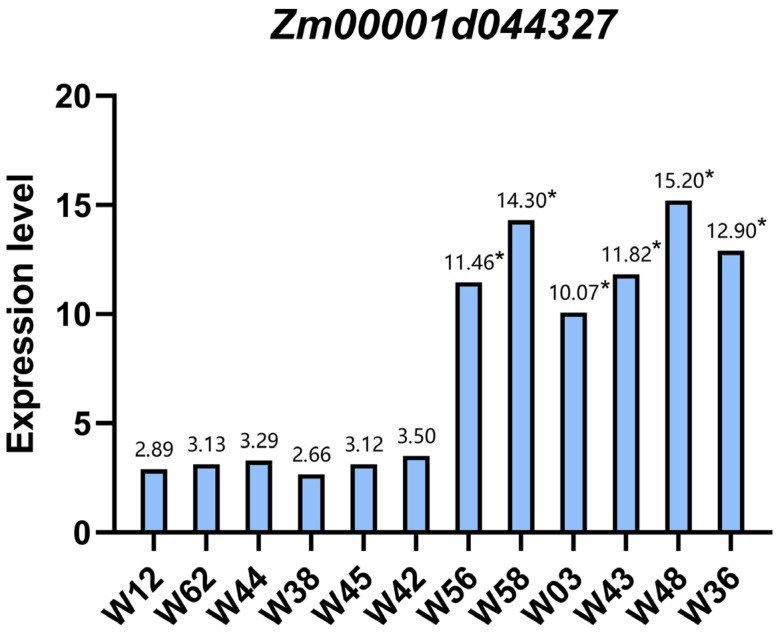
*Zm00001d044327* qPCR expression levels. ‘*’ indicates statistical significance at *p* < 0.05 (Student’s *t*-test).

Through this approach, we found that *Zm00001d044327* was expressed at significantly higher levels in inbred maize lines with broad leaves (W12, W62, W44, W38, W65, and W42) (*p* < 0.05) relative to those with narrow leaves (W56, W58, W3, W43, W48, and W36) (Figure 5).

### 3.7. Assessment of the Phenotypic Impact of Zm00001d044327 Candidate Gene Mutations 

**Figure 6:**
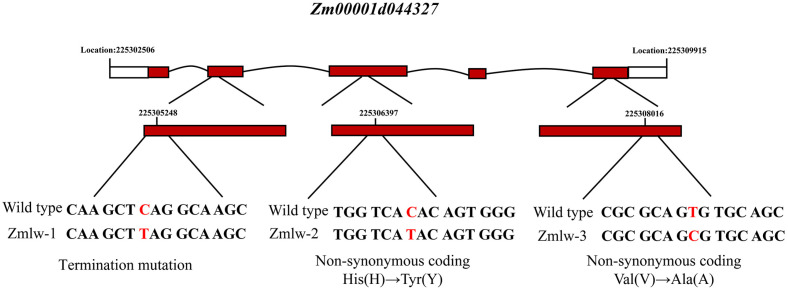
*Zm00001d044327* mutant position and formation.

**Figure 7:**
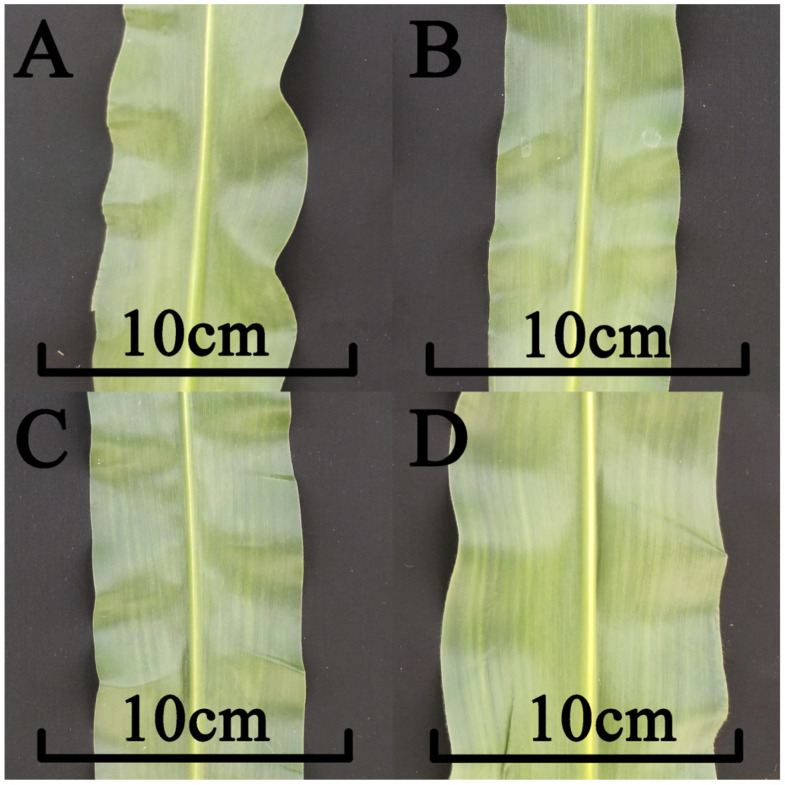
*Zm00001d044327* mutant leaf width. (**A**) Mutants ZMLW-1 leaf width; (**B**) mutant ZMLW-2 leaf width; (**C**) mutant ZMLW-3 leaf width; (**D**) wild-type B73 leaf width.

Next, analyses were conducted based upon EMS mutants with a conserved amino acid termination mutation and two non-synonymous mutations in *Zm00001d044327*. Zmlw-1 was a conserved amino acid termination mutation (mutation location: chr3, 225305248). Zmlw-2 (mutation location: chr3, 225306397) and Zmlw-3 (mutation location: chr3, 225308016) are two non-synonymous mutants in the LOG domain of *Zm00001d044327*, with Zmlw-2 causing a non-synonymous tyrosine (Y) substitution for a histidine (H) and Zmlw-3 causing a non-synonymous arginine (A) substitution for a valine (V) (Figure 6). Relative to the wild-type inbred B73 maize line, the leaf width values for these three mutants were significantly narrower (Table 3, Figure 7).

## 4. Discussion

### 4.1. Leaf Width-Related SNP Markers

In this study, we utilized the farmCPU genome-wide association strategy to mitigate the confounding effects of mixed genetic backgrounds and reduce false-negative outcomes [26]. This method facilitated a more precise identification of single nucleotide polymorphisms (SNPs) associated with leaf width in inbred maize lines. Notably, several detected SNPs, such as sLW-141511859 and sLW-104304481, were localized within the chromosomal regions bin5.04 and bin7.02, respectively. These regions correspond to previously characterized quantitative trait loci (QTLs) associated with leaf width, specifically Y168871-umc1687 as identified in Tang’s research [27]. Additionally, our results corroborated findings from Zhang et al., where sLW-30006587 and sLW-11019663 SNPs aligned with bnlg1808-dupssr9 and bnlg2235-bnlg162 QTLs [28]. Furthermore, the sLW-226131901 SNP was concordant with the phi101049-bnlg1520 QTL, and the sLW-225305520 SNP matched the umc1608-umc1030 QTL identified in a Yu82 × Shen137 F2:3 population analysis conducted by Ku et al. [29]. Our analyses also unveiled novel SNP markers potentially linked to leaf width, underscoring the complex multigenic inheritance of this trait. Challenges in precise microgene localization were noted, reflecting limitations in the parental QTL mapping strategies employed in prior studies. 

### 4.2. Candidate Gene Functional Analysis

In our genome-wide association study, we identified the candidate gene *Zm00001d044327*, which harbors a significant SNP associated with leaf width. Notably, this gene was not covered in our previous research published in PLOS One [10], highlighting a novel genetic component in the architecture of maize leaf width. This gene is annotated to encode cytokinin phosphoribohydrolase LOG7 in the TAIRP43 database. Given that this enzyme is integral to cytokinin biosynthesis and metabolism [30], it is hypothesized that LOG7 influences leaf width through the modulation of hormone synthesis and signaling pathways. Expression analysis revealed that *Zm00001d044327* is predominantly expressed in maize foliage, reaching peak expression in the ninth leaf (V9). Notably, expression levels were elevated in broad-leaved phenotypes compared to their narrow-leaved counterparts. Ethyl methanesulfonate (EMS) mutagenesis confirmed the gene’s role, where structural alterations in *Zm00001d044327* correlated with reduced leaf width above the uppermost ear, substantiating its regulatory function in leaf morphology.

Further investigations led to the identification of *Zm00001d020287*, located 2528 bp upstream of SNP sLW-104303853. This gene encodes the hypothetical protein ZEAMMB73, implicated in plant-bacterial interactions [31]. Additionally, the gene *Zm00001d007267*, positioned 9706 bp from sLW-226131901, encodes the chlorophyll a-b binding protein. This protein plays a pivotal role in the photosynthetic apparatus, facilitating the transduction of light signals within maize foliage [32].

## 5. Conclusions

We performed a GWAS-based assessment of 80 backbone inbred maize lines and 1.49 × 10^6^ SNPs, leading to the identification of 14 SNPs significantly associated with leaf width based upon two years of phenotypic data (*p* < 0.000001), with these SNPs accounting for 14.2% of the observed phenotypic variability in this trait. The sLW225305520 marker was localized to an exonic region in the *Zm00001d044327* candidate gene, resulting in the nonsynonymous substitution of leucine (L) in place of phenylalanine (F) at amino acid position 227. 

In total, five potential leaf width-associated genes were identified within the LD region. Of these genes, *Zm00001d044327*, which encodes a cytokinin phosphoribohydrolase, was of particular interest as it was predicted to positively regulate leaf width, it was expressed at higher levels in broad-leaved plants relative to narrow-leaved plants, and it harbored a significant SNP detected through this GWAS approach. The relevance of this candidate gene was further confirmed based upon analyses of EMS mutants with one conserved amino acid termination mutation and two non-synonymous mutations in *Zm00001d044327*, as these plants exhibited a narrow leaf width variation.

Together, our data provide a robust scientific basis for future studies pertaining to the genetic control of maize leaf width, while also providing a theoretical foundation for efforts to optimize maize leaf width properties through accelerated molecular-based breeding strategies.

## Figures and Tables

**Table 1:** qPCR primers.

Primer	Sequence (5′-3′)	Candidate Gene
QLWS	TGCTCAGTCAGCAGCATATC	*Zm00001d044327*
QLWAS	CCTTCCTTCCTCCCTACTTCTA	

**Table 2:** Significant SNPs and candidate genes associated with leaf width in 2015 and 2016.

Year	SNP	Physical Position	Chr	Genotype	MAF	log10 (P)	Distance (bp)	Contribution (r2)	Candidate Gene	Functional Annotation
2015	sLW-226131901	226131901	2	A/G	0.05	6.31	−9706	6.20%	*Zm00001d007267*	Chlorophyll a-b binding protein CP26, chloroplastic
	sLW-225322565	225322565	3	C/T	0.07	6.33		6%		
	sLW-225305520	225305520	3	C/T	0.30	6.35	0	13.20%	*Zm00001d044327*	Cytokinin riboside 5′-monophosphate phosphoribohydrolase LOG7
	sLW-141511859	141511859	5	G/A	0.11	6.11		4.90%		
	sLW-150065211	150065211	6	T/A	0.06	6.07		0.40%		
	sLW-30006587	30006587	7	T/G	0.34	6.73		3.20%		
	sLW-104304481	104304481	7	C/A	0.05	6.17	3128	7.10%	*Zm00001d020285*	Putative pumilio homolog 7, chloroplastic
	sLW-104304481	104304481	7	C/A	0.05	6.17	−2528	7.10%	*Zm00001d020287*	hypothetical protein ZEAMMB73
	sLW-68332493	68332493	8	C/T	0.12	6.04		1.90%		
2016	sLW-20566973	20566973	1	G/T	0.07	8.60		3.10%		
	sLW-186867035	186867035	2	C/A	0.07	12.81	2768	13.60%	*Zm00001d005758*	
	sLW-153643898	153643898	5	G/T	0.13	8.38		14.20%		
	sLW-11019663	11019663	8	C/T	0.49	9.26		7.20%		
	sLW-12911742	12911742	9	T/A	0.10	13.09		3.70%		
	sLW-100159475	100159475	9	T/A	0.13	10.23		11.60%		

**Table 3:** Mutant leaf width phenotype variations.

Mutant	Leaf Width above the Uppermost Ear (cm)	Sig.
wild type (B73)	9.8	A
ZMLW-1	7.2	B
ZMLW-2	6.8	B
ZMLW-3	7.4	B

## Data Availability

All data supporting the findings of this study are available within the paper. Raw GWAS data used in this study have been deposited at the NCBI SRA database under accession number PRJNA495031.
